# Non-classical HLA-E restricted CMV 15-mer peptides are recognized by adaptive NK cells and induce memory responses

**DOI:** 10.3389/fimmu.2023.1230718

**Published:** 2023-09-21

**Authors:** Nerea Martín Almazán, Benedetta Maria Sala, Tatyana Sandalova, Yizhe Sun, Tom Resink, Frank Cichocki, Cecilia Söderberg-Nauclér, Jeffrey S. Miller, Adnane Achour, Dhifaf Sarhan

**Affiliations:** ^1^ Department of Laboratory Medicine, Division of Pathology, Karolinska Institute, Stockholm, Sweden; ^2^ Science for Life Laboratory, Department of Medicine, Karolinska Institute, Stockholm, Sweden; ^3^ Division of Infectious Diseases, Karolinska University Hospital, Stockholm, Sweden; ^4^ Division of Hematology, Oncology and Transplantation, University of Minnesota Masonic Cancer Center, Minneapolis, MN, United States; ^5^ Department of Medicine, Microbial Pathogenesis Unit, Karolinska Institute, Stockholm, Sweden; ^6^ Division of Neurology, Karolinska University Hospital, Stockholm, Sweden; ^7^ Institute of Biomedicine, Unit for Infection and immunology, MediCity Research Laboratory, InFLAMES Flagship, University of Turku, Turku, Finland

**Keywords:** Adaptive NK cells, memory, dendritic cells, peptides, HLA-E

## Abstract

**Introduction:**

Human cytomegalovirus (HCMV) reactivation causes complications in immunocompromised patients after hematopoietic stem cell transplantation (HSCT), significantly increasing morbidity and mortality. Adaptive Natural Killer (aNK) cells undergo a persistent reconfiguration in response to HCMV reactivation; however, the exact role of aNK cell memory in HCMV surveillance remains elusive.

**Methods:**

We employed mass spectrometry and computational prediction approaches to identify HLA-E-restricted HCMV peptides that can elucidate aNK cell responses. We also used the K562 cell line transfected with HLA-E0*0103 for specific peptide binding and blocking assays. Subsequently, NK cells were cocultured with dendritic cells (DCs) loaded with each of the identified peptides to examine aNK and conventional (c)NK cell responses.

**Results:**

Here, we discovered three unconventional HLA-E-restricted 15-mer peptides (SEVENVSVNVHNPTG, TSGSDSDEELVTTER, and DSDEELVTTERKTPR) derived from the HCMV pp65-protein that elicit aNK cell memory responses restricted to HCMV. aNK cells displayed memory responses towards HMCV-infected cells and HCMV-seropositive individuals when primed by DCs loaded with each of these peptides and predicted 9-mer versions. Blocking the interaction between HLA-E and the activation NKG2C receptor but not the inhibitory NKG2A receptor abolished these specific recall responses. Interestingly, compared to the HLA-E complex with the leader peptide VMAPRTLIL, HLA-E complexes formed with each of the three identified peptides significantly changed the surface electrostatic potential to highly negative. Furthermore, these peptides do not comprise the classical HLA-E-restriction motifs.

**Discussion:**

These findings suggest a differential binding to NKG2C compared to HLA-E complexes with classical leader peptides that may result in the specific activation of aNK cells. We then designed six nonameric peptides based on the three discovered peptides that could elicit aNK cell memory responses to HCMV necessary for therapeutic inventions. The results provide novel insights into HLA-E-mediated signaling networks that mediate aNK cell recall responses and maximize their reactivity.

## 1 Introduction

Natural Killer (NK) cells are innate immune cells that mediate immune responses against intracellular pathogens and cancer. NK cells do not require prior activation or binding of a specific antigen. Instead, their activation is regulated mainly by a range of inhibitory and activating receptors (1, 2). Nevertheless, a growing number of experimental and clinical studies support the unique role of a subpopulation of NK cells termed adaptive (a)NK cells in possessing an elevated specific response to viral peptides through ligation of the non-polymorphic HLA-E to the activation receptor NKG2C (3, 4). However, little is known concerning the antigen presentation mechanisms underlying the recognition of viral peptides by aNK cells.

It has been demonstrated during the last decade that aNK cells, which have virus-specific immunological memory, accumulate in human cytomegalovirus (HCMV)-infected individuals and recognize peptides derived from HCMV-encoded proteins through HLA-E. These include the UL40 molecule that contains a nonameric epitope with an identical sequence to endogenous HLA-E-binding peptides (3, 5, 6). HCMV is a widespread virus, a member of the Beta-herpesviridae family, that infects 60-90% of the adult population. After a primary infection, the virus establishes life-long latency in its host (7). HCMV has developed multiple immune evasion strategies to avoid and hamper immune responses (8). HCMV has also profound effects on NK cell phenotype, proliferation, and function (9). The unique aNK cell subset was first described in association with the response to HCMV infection. These aNK cells are similar to mature conventional (c) NK cell populations and express CD57, the activating receptor NKG2C, while also displaying downregulation of the inhibitory counterpart NKG2A that binds to HLA-E. aNK cells have common epigenetic signatures resulting in the downregulation of the transcription factor PLZF and the proximal signaling molecules SYK, EAT-2, and FcεR1γ (10, 11). These epigenetic changes have been shown to persist long-term *in vivo* (12). aNK cells have been hypothesized to go through the same memory phases as T cells (clonal expansion, contraction phase, and memory formation). Furthermore, similarly to CD8^+^ T cells, aNK cells go through mitophagy, which is itself a hallmark of immunological memory formation (13).

HCMV encodes a large number of different proteins, including the tegument protein pp65 and the immediate early protein (IE), that modulate immune responses of NK cells, as well as T and dendritic cells (DC) (14, 15). Similar to T cells, NK cells may also be primed by DCs and enhances NK cell responses (16, 17). Also, aNK cell accumulation in HCMV-seropositive individuals indicates that pp65 may not inhibit the proliferative capacity and the functional activity of aNK cells (18).

HCMV reactivation is associated with adverse clinical outcomes in immunosuppressed individuals, such as post hematopoietic stem cell transplantation (HSCT) patients, and occurs in up to 70% of such HCMV-seropositive recipients (19). HCMV reactivation takes place in severely ill immunocompetent patients and is associated with prolonged hospitalization or death (20). Effective HCMV prophylaxis is warranted for both immunocompromised and immunocompetent patients. We have previously shown that aNK cell presence is associated with anti-tumor effects, reduced relapses, and better clinical responses following HSCT, as well as resistance to suppressive cells in the tumor microenvironment (21–24). Thus, it is highly important to develop a long-lasting and efficient memory response in aNK cells towards HCMV. This could represent one of the best strategies to prevent graft rejection and relapse over time, allowing us to control HCMV reactivation.

In this study, we hypothesized that, similar to T cells, DCs can present pathogenic peptides to aNK cells. We refolded HLA-E with an ensemble of 15-mer overlapping peptides that cover the entire length of the HCMV protein pp65. Using mass-spectrometry, we e discovered three HLA-E binding pp65-derived peptides that provoke aNK cell memory responses specifically towards HCMV. Our results demonstrate that aNK cells require recognition of HLA-E-restricted peptides presented by DCs that depends on NKG2C and HLA-E interaction, resulting in a significant aNK cell expansion with enhanced capacity to recognize and kill HCMV-infected target cells. Our findings pave the way for new and novel therapeutic inventions that could potentially limit the clinical severe complications caused by HCMV reactivation.

## 2 Materials and methods

### 2.1 Blood donors

Peripheral blood mononuclear cells (PBMCs) from healthy HCMV-seropositive or seronegative donors were obtained from Memorial Blood Bank (Minneapolis, MN) and the Stockholm blood bank. All participants gave informed consent to participate in the study before taking part. All samples were de-identified before receipt and approved for use by the university institutional review board in accordance with the Declaration of Helsinki.

### 2.2 Cell isolation

PBMCs were isolated from buffy coats by density gradient centrifugation using Ficoll-Paque Premium (GE Healthcare). Monocytes were isolated by positive selection using anti-CD14 microbeads (Miltenyi Biotech). Untouched CD3-CD56+ NK cells were isolated using negative selection kits (Miltenyi Biotech). NK cell donors with ≥ 4% aNK cells were used for further analysis.

### 2.3 Identification of peptide sequences through mass spectrometry

A pool of 138 different 15-mer peptides covering the sequence of full-length pp65, kindly provided by the NIH AIDS reagent program, was refolded with HLA-E and human β2-microglobulin (hβ2m) as previously described (25, 26). The obtained HLA-E/hβ_2_m/peptide complexes were isolated using size-exclusion chromatography. Peptides were thereafter eluted from the purified MHC/peptide complexes under acidic conditions (0.1% trifluoroacetic acid, 10% CH3CN), further purified using a 5 Kd Ultrafree-15 centrifugal filter device (Millipore) and concentrated using speed vac (Labconco). All experiments were repeated independently three times. The sequences of all eluted peptides were analyzed using mass spectrometry. Peptide sequences detected in at least two independent experimental replicates were further sent for synthetization. Furthermore, a selection of nonameric peptides predicted through the molecular modeling of the 15-mer in complex with HLA epitopes identified by MS were also produced. The 9-mer sequences were predicted based on each non-conventional residue’s ability to fit the HLA-E peptide binding cleft.

### 2.4 Cell culture

CD14^+^ monocytes (M), purified from HCMV-seropositive and -seronegative individuals, were differentiated into immature DC (imDC) in GM-CSF (100 ng/ml) and IL-4 (20 ng/ml) for three days (2x10^6^/ml in 2 ml/well, 6-well plates). Later, imDC were washed, counted and re-cultured (1x10^6^/ml in 6-well plate) overnight in DC-CellGro® medium (Cellgenix), supplemented with 2% human AB-serum and differently conditioned; imDC: GM-CSF (100 ng/ml) and IL-4 (20 ng/ml); mature DC (mDC): GM-CSF (final 100 ng/ml), IL-4 (final 20 ng/ml), IFNg (1000 IU/ml), TLR7/8 agonist R848 (2.5 ug/ml), poly IC (20 ug/ml), LPS (10 ng/ml) (Peprotech and Sigma Aldrich), and in the presence or absence of customized HLA-E-binding peptides identified within this study, and other control and a selection of previously known HLA-E-restricted peptides (**Table 1**, Nordic Biosite, and NIH AIDS reagent program IEDB). A control experiment was performed culturing IL-15-activated NK cells with MRC-5 fibroblast cell line (ATCC) in the presence or absence of pp65 and accessed for aNK cell activities.

**Table 1:** Peptides used in the study and melting temperature measured with nanoDSF.

Sequence	Name	Melting Temperature (Tm)
**RGPGRAFVTI**	P18-I10, H-2D^d^-restricted control peptide	
**QMRPVSRVL**	hsp60, HLA-E-restricted control peptide	
**VMAPRTLIL**	UL40, HLA-E-restricted control peptide	
**The pool of 129 HIV overlapping 15mer-peptides**	All 15mers are derived from the HIV-1 protein Gag	
**The pool of 138 overlapping 15mer-peptides**	All 15mers are derived from the HCMV protein pp65	
**SEVENVSVNVHNPTG^1^**	pp65_85-99_ MS-identified 15mer	62.1°C
**TSGSDSDEELVTTER^2^**	pp65_401-415_ MS-identified 15mer	62.6°C
**DSDEELVTTERKTPR ^3^**	pp65_405-419_ MS-identified 15mer	52.6°C
**EVENVSVNV**	pp65_402-410_; 9-mer predicted from ^1^	62.1°C
**SGSDSDEEL**	pp65_86-94_; 9-mer predicted from ^2^	61.9°C
**VTTERKTPR**	pp65_411-419_; 9-mer predicted from ^3^	53.7°C
**DSDEELVTT^4^**	pp65_405-413_; 9-mer predicted from ^2^ and ^3^; Higher binding affinity to HLA-E	54.6°C
**DMDEELVLL**	pp65_405-413_(p2M, p9L); Altered peptide ligand version from ^4^ with predicted higher binding affinity to HLA-E	54.1°C
**DQDEELVTL**	pp65_405-413_(p2Q, p9L); Altered peptide ligand version from ^4^ with predicted higher binding affinity to HLA-E	54.9°C

Underlined: HLA-E restricted motif.Superscript: The 15-mer peptides reference.

Following overnight maturation, DC were washed, counted, and co-cultured with NK cells in 96-well plates at a concentration of 0.05-0.15 x 10^6^ cells and 1:10 ratio per well in RPMI-1640 with 10 ng/ml IL-15 for 14 days. In further experiments, NK cells were cultured with mDC+pp65-peptides for 14 days in addition (5 μg/ml) of either a control isotype-matched antibody IgG (clone Poly4053), anti-NKG2C (clone 134522), anti-NKG2A (clone 131411), or anti-HLA-E (clone 3D12), anti-MHC I (clone W6/32), and MHC II (Tü39) blocking antibodies (27–30) (BioLegend, R&D systems) at the primary phase (day 0 and 7) or at the secondary restimulation phase (at day 14, during 6h stimulation).

### 2.5 Flow cytometry

Cells were stained with fluorochrome-conjugated antibodies against the following antigens: CD14, CD80, CD83, HLA-E, CD56, CD3, CD57, NKG2C, NKG2A, CD45RA, CD45RO, FcεRIγ/Syk, IFN-γ, Ki67 (proliferation), CD107a (degranulation), and TNFα, all from Biolegend. All staining was performed in combination with Live/Dead Fixable Dead Cell Stain (Thermo-Fisher) to exclude dead cells. Detection of intracellular FcεRIγ/Syk, IFN-γ, Ki67 (proliferation), CD107a (degranulation), and TNFα was performed following fixation and permeabilization (eBioscience) according to the manufacturer’s instructions. Cells were acquired on either an LSRII or Fortessa cytometer (BD Biosciences) and data were analyzed using FlowJo (TreeStar) and Cytobank Premium (Beckman Coulter) (31).

### 2.6 NK cell degranulation and cytokine production assays

For determination of NK cell cytolytic activity (degranulation), as well as IFN-γ and TNFα production, cells were incubated at 37°C for 6 hours at a 1:1 ratio with either the MRC-5 cells that were pre-infected with the HCMV strain VR1814 at MOI 1 for three days, or with uninfected MRC-5 cells followed by flow cytometry analysis. CD107a, GolgiPlug, and GolgiStop (BD Biosciences) were added to the culture media during incubation. Intracellular staining and flow cytometry analyses were then performed.

### 2.7 NK cell killing assays

To determine NK cell killing capacity, HCMV-infected or uninfected MRC-5 cells were fluorescently labeled with CellTrace Violet (5 uM, Invitrogen), and target killing was evaluated using Live/Dead dye (Invitrogen) following a six-hour incubation at an effector to target (E: T) ratio of 1:1. MRC-5 cell killing was assessed by gating on CD45 negative populations, further gated on CellTrace positive population and assessed for the proportion of Live/Dead^+^ cells.

### 2.8 HLA-E/peptide production and purification

The HLA-E*0101 heavy chain and hβ_2_m were expressed individually as inclusion bodies using the BL21 (DE3) *E. coli* strain, following previously published protocols (25, 26). Inclusion bodies were solubilized in 8 M Urea, 100 mM Tris HCl pH 8, and 2 mM EDTA. The refolding of HLA-E/peptide complexes was carried out by the following dilution: 3 mg of peptide and 8 mg of hβ2m were added firstly to the refolding buffer (100 mM Tris pH 8, 450 mM L-Arginine, 5 mM L-Glutathione reduced, 0.5 mM L-Glutathione oxidized, 2 mM EDTA, 0.5 mM AEBSF) and the solution was left at 4°C under stirring for half an hour. 4 mg of unfolded HLA-E was then added in three steps. After 24 hours, the refolding solution was concentrated to approximately 5 mL. The sample was then purified by size exclusion chromatography using a HiLoad 16/60 Superdex 200 pg column equilibrated with 20 mM Tris HCl pH 8 and 150 mM NaCl. The eluted protein was analyzed by SDS-PAGE, frozen in liquid nitrogen, and stored at -20°C.

### 2.9 Nano differential scanning calorimetry (NanoDSF)

Thermal unfolding experiments were performed by nanoscale differential scanning fluorimetry (nanoDSF) (32). The protein intrinsic fluorescence during the thermal ramp was followed at 330 nm and 350 nm with a Prometheus NT.48 instrument from NanoTemper Technologies with an excitation wavelength of 280 nm 28. Capillaries were loaded with 10 ul of protein at a concentration of 1 mg/mL in 20 mM Tris-HCl, pH 8.0, and 150 mM Sodium Chloride. The temperature ramp measurements were recorded from 20 to 95°C (temperature slope 60°C/hour). Three independent measurements were carried out for each complex. The fluorescence intensity ratio was recorded, and its first derivative was calculated with the manufacturer’s software (PR.ThermControl, version 2.1.2).

### 2.10 Molecular modeling of HLA-E/peptide complexes

The molecular modeling of the three-dimensional structures of HLA-E/peptide complexes was performed using the crystal structure of HLA-E/CD94/NKG2A (33) (PDB code 3CDG) as a preliminary template and assuming that CD94/NKG2C similarly interacts with HLA-E to CD94/NKG2A. The modeling was performed manually in the program Coot (34), followed by model regularization to improve the peptide chain’s geometry and remove all possible sterical hindrances. Flanking peptide residues were modeled in an arbitrary conformation to demonstrate that HLA-E can present longer peptides and do not prevent CD94/NKG2C binding. Peptide elongation at the C- and N-terminal of the HLA-E peptide binding cleft was possible using different rotamers for the side chains or residues K146 and W167, respectively. None of the introduced flanking peptide residues interact with the CD94/NKG2C heterodimer.

### 2.11 HLA-E binding assay

Peptide binding assay was performed in the HLA-E*0101 transfected K562 cell line (K562E*0101), kindly provided by Dr. Jakob Michaelsson (Karolinska Institutet, Center for Infection Medicine, Department of Medicine, Huddinge), and mycoplasma tested before use. These K562E*0101 cells have a constitutive HLA-E expression. Briefly, cells were re-suspended in a medium at 10^6^ cells/ml, and indicated peptides were added at a concentration titration of 0-100 µM. After an overnight incubation at 37°C, cells were washed with PBS to remove free peptides. Next, HLA surface expression was monitored after staining with anti-HLA-E (BioLegend) and viability dye. Analysis was done using flow cytometry as described above. Results are reported as flow cytometry histograms or mean fluorescence intensity (MFI) compared to Fluorescence Minus One (FMO) control.

### 2.12 Data management and statistical analysis

All experiments were repeated independently at least three times. One representative and accumulative data are presented. All numeric data were subjected to a normal distribution test before further statistical analysis. For the comparison within groups, parametric or non-parametric multiple comparison two-way ANOVA or one-way ANOVA tests were performed. Student’s T-test was used when comparing two groups only. All statistical tests were two-sided and ± SEM. All p-values or asterisks from multiple comparisons were corrected using the FDR method <0.05. No asterisk or p-values represent not significant data. The Prism v9.2 software (GraphPad) was used for statistical analyses. All dimensional reduction opt-SNE analyses based on flow cytometry data were done utilizing the Cytobank 29 cloud-based platform.

## 3 Results

### 3.1 aNK cells expand significantly following co-culture with mature DCs that present a pool of HCMV pp65-derived 15-mer peptides

**Figure 1:**
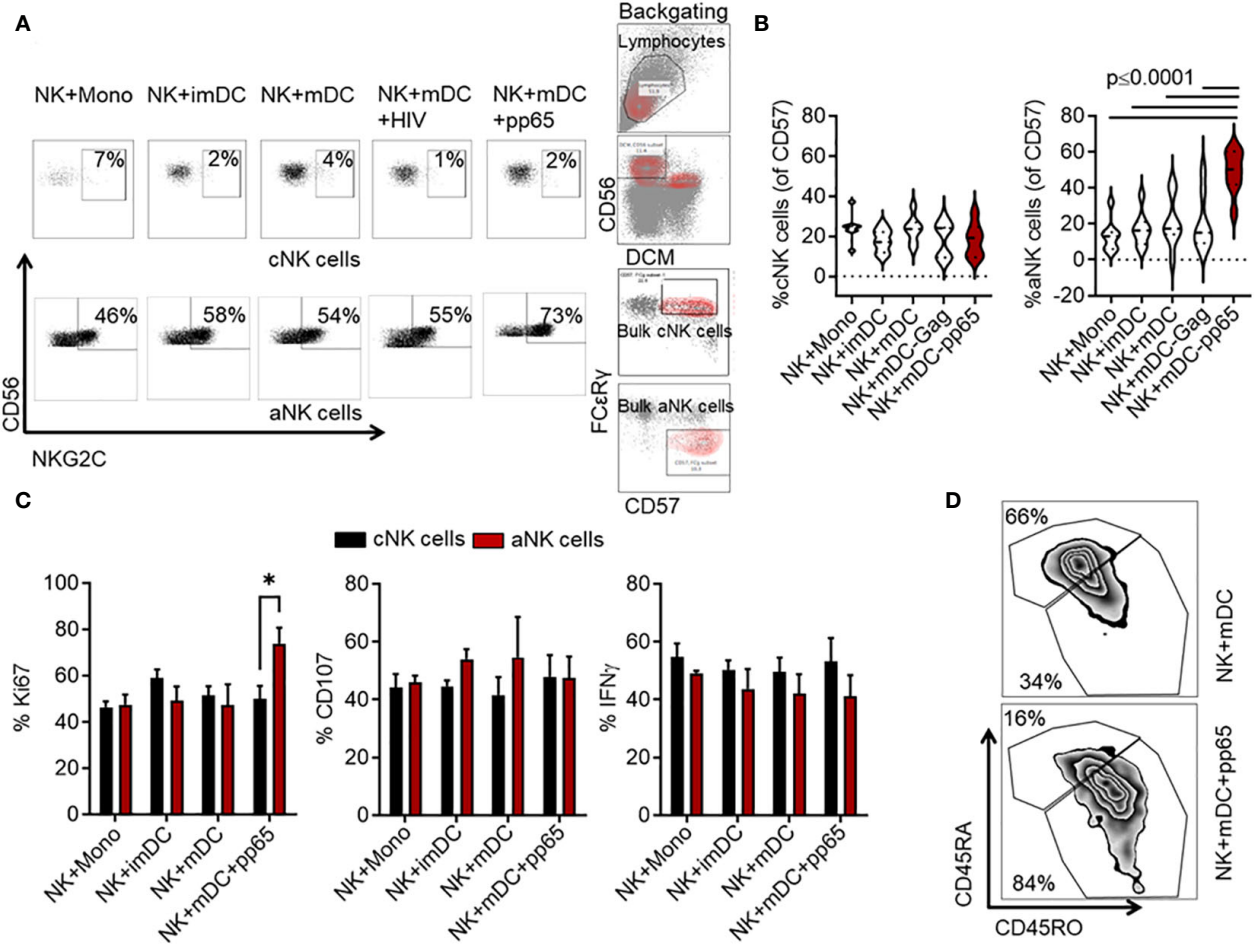
Co-culture of mDC loaded with an HCMV-associated pp65-derived peptide pool significantly expands the frequency of aNK cells. NK cells from HCMV-seropositive donors were cultured for 14 days with mDC loaded with the pools of overlapping 15-mer peptides derived from either HCMV-associated pp65 or HIV-1-associated Gag proteins, or left unloaded in the presence of 10 ng/ml IL-15 relative to cocultures with monocytes and imDC. **(A)** Representative flow cytometry plots and gating strategy of CD56/NKG2C cNK and aNK cell expansion are shown. **(B)** Cumulative (n = 10) data showing the percentages of cNK and aNK cells within the total CD57+ NK cell population. Results from five independent experiments are presented as mean ± SEM. A One-way ANOVA test was used for statistical analyses. **(C)** Cumulative (n = 9) data showing the percentages of aNK and cNK cell proliferation (Ki67), degranulation (CD107a), and IFNγ production following co-culture with M, imDCs, mDCs, or mDCs+pp65. The presented results are from three independent experiments. All the cumulative data are shown as mean ± SEM. A two-way ANOVA test was used for statistical analyses. **(D)** Representative data from three independent experiments showing NK cell phenotype based on CD45RA and CD45RO expression levels when in co-culture with mDC or mDC+pp65. * indicating p-values ≤ 0.05.

A previous study revealed that NKG2C^+^ NK cells specifically recognize distinct HCMV strains that encode a heterogeneous repertoire of the classical UL40 peptides. These peptides control the expansion and differentiation of NKG2C^+^ aNK cells (3). However, that study did not reveal whether other HCMV-derived peptides can control aNK cell recall responses. Neither did previous studies investigate the involvement of professional antigen-presenting cells (APCs), such as DCs, in priming human aNK cells. Here, we first assessed the recognition specificity of aNK cells towards a large array of overlapping HCMV 15-mer peptides derived from the pp65 protein. Purified NK cells from HCMV-seropositive individuals were co-incubated at a 10:1 ratio with autologous monocytes or imDC representing poor APCs and mDC as professional APC, either loaded with pools of 15-mer peptides derived from the HCMV-associated pp65 protein or unloaded. As a control, we used a control pool of peptides derived from the HIV-associated Gag protein. This high-throughput phenotypic screening allowed us to identify HCMV-specific expansion of aNK cells. Interestingly, only the addition of mDC loaded with the pp65-peptide pool (referred to as mDC^+^pp65) to the NK cell culture led to a consistent increase in the NKG2C^+^CD56^+^ aNK cell pool (CD3^-^CD56^+^CD57^+^FCεRγ^-^) (p ≤ 0.03). In contrast, cNK cell frequency (CD3^-^CD56^+^CD57^+^FCεRγ^+^) did not increase, relative to all other controls (**Figures 1A, B**). Complementing this observation, we found a significant increase in the proliferation index of aNK cells (% Ki67) compared to cNK cells following the addition of mDC^+^pp65, which was not seen in the other tested conditions (73.9 ± 12, p= 0.04) (**Figure 1C**). However, we were unable to establish any functional difference, including degranulation and cytokine production between cNK and aNK cells following generic stimulation with an agonistic anti-CD16 antibody combined with IL-12 and IL-18 recombinant cytokines, suggesting a need for a specific secondary stimulation to induce functional advantage in aNK cells. Further analysis revealed that compared to NK cells cocultured with peptide unloaded control DC, NK cells co-cultured with mDC^+^pp65 displayed a remarkable increase in the CD45RO population, which has been shown to represent mature and functional NK cells in hematological malignancies and identify memory NK cells (35, 36) (**Figure 1D**). Our results suggest that peptide priming stimulates and generates memory-like NK cells.

### 3.2 Peptide recognition by aNK cells is dependent on both HLA-E and NKG2C

**Figure 2:**
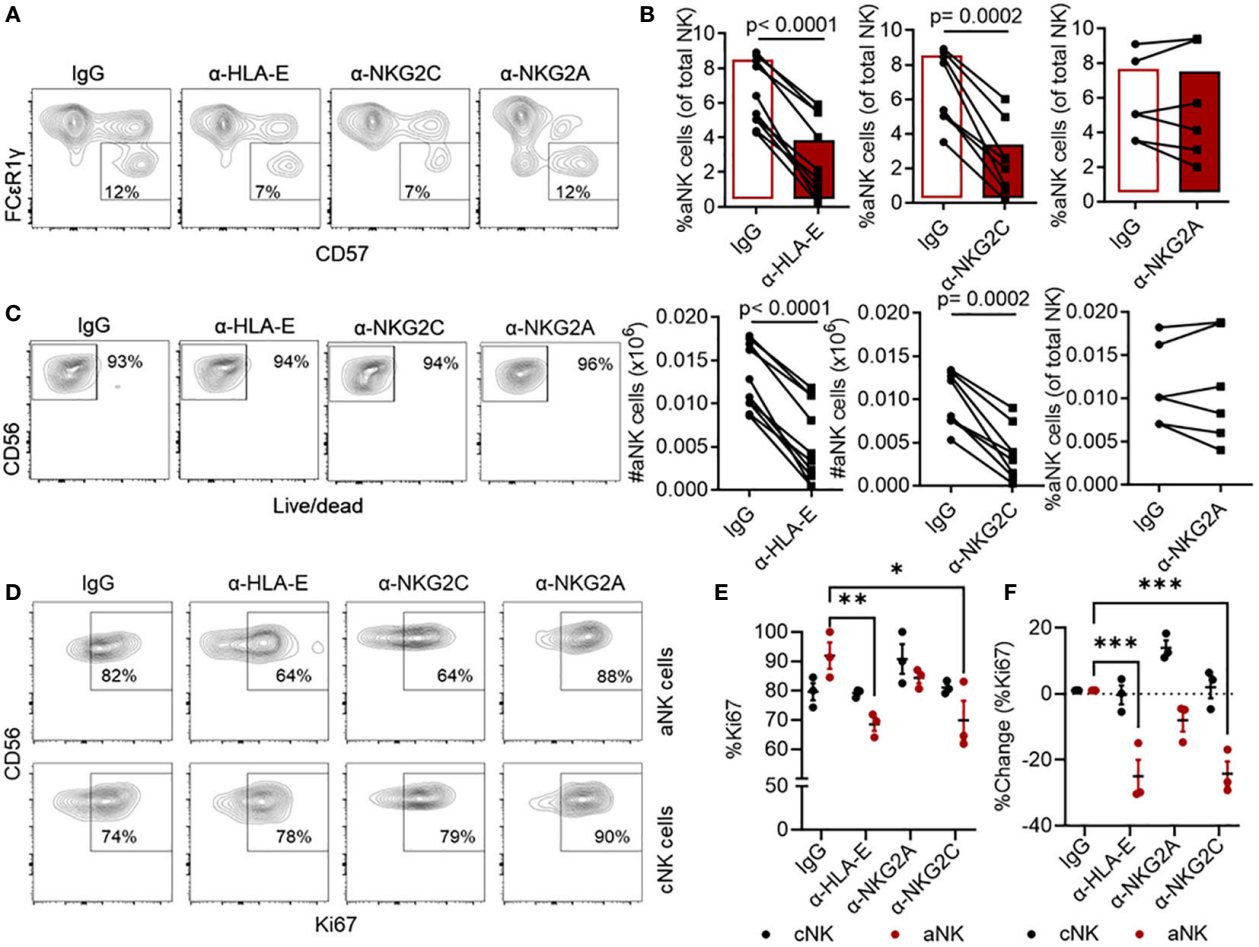
aNK cell proliferation is dependent on HLA-E/NKG2C interactions. **(A)** Representative gating strategy of NK cells (n = 6-10) cultured with mDC+pp65 for 14 days in the presence of either a control isotype-matched antibody IgG, or anti-HLA-E, anti-NKG2C, or anti-NKG2A blocking antibodies (5 μg/ml), thereafter assessed for their **(B)** (top panel) frequency and (bottom panel) number of cells, **(C)** viability, and **(D, E)** proliferation by flow cytometry. All blood donors were HCMV seropositive. **(F)** Percentage change (blocking – IgG) from **(E)** is shown. The results from 2-5 independent experiments are presented, and data are shown as representative plots or for each donor, where the donor in **(A, C)** is the same, and the donor in **(D)** is included in accumulative data in **(E)**. Student’s T-test and Two-way ANOVA were used for statistical analyses. * indicating p-values ≤ 0.05, ** indicating p-values ≤ 0.001, *** indicating p-values ≤ 0.0001.

Earlier studies have demonstrated the importance of interactions between NKG2C and HLA-E molecules in NK cell responses to HCMV (3, 28). It is also well-established that NKG2A is an inhibitory receptor, expressed on both NK and T cells, that competes with the activating receptor NKG2C for HLA-E binding (37, 38). Our previous studies revealed that aNK cells have low or no NKG2A expression, making them less susceptible to NKG2A-mediated inhibition (10, 22). Here, we investigated whether aNK cell responses to mDC^+^pp65 dependent on the interaction of HLA-E with any of these two NK cell receptors. For this purpose, NK cells were cultured with mDC^+^pp65 in the presence of blocking antibodies specific to either HLA-E (39), NKG2C (40), NKG2A (41), or a control IgG isotype. Our results demonstrated that antibody blocking of HLA-E or NKG2C abolished the expansion of aNK cell population, observed in response to stimulation by mDC^+^pp65 and the presence of control IgG antibodies. In contrast, we did not observe any difference in the expansion of aNK cells when blocking the NKG2A receptor interaction, confirming that aNK cell responses are independent of NKG2A, instead dependent on the interaction between HLA-E- HCMV- peptide complexes and NKG2C (**Figures 2A, B**). The reduction in expansion of aNK cells in the presence of anti-HLA-E or anti-NKG2C antibodies was not due to changes in cell viability, rather reflected a lack of specific proliferation (**Figures 2C–F**). In control experiments, we confirmed the blocking capacity of these antibodies. Using the K562 cell line transfected with HLA-E and loaded with pp65 peptide pool and cocultured with NK cells; we found that aNK cell degranulation was reduced when blocking NKG2C and HLA-E. On the other hand, assessing cNK cell degranulation, we found that blocking NKG2A enhanced their degranulation capacity (**Supplementary Figures 1A, B**). Thus, these results indicate substantial participation of NKG2C and HLA-E in the aNK cell antigen priming phase by DCs.

### 3.3 Identification of three pp65-derived HLA-E-restricted 15-mer epitopes that do not comprise the classical motif

**Figure 3:**
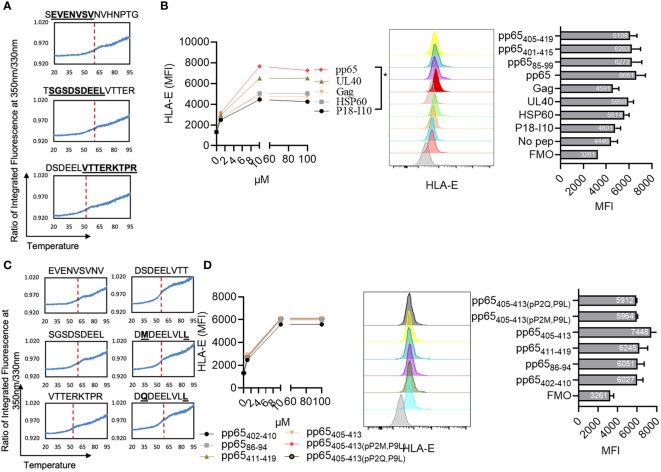
Identification of three 15-mer and 9-mer pp65-derived HLA-E-restricted epitopes. **(A)** NanoDSF studies to assess the thermal stability of HLA-E in complex with the following peptides: SEVENVSVNVHNPTG, DSDEELVTTERKTPR, and TSGSDSDEELVTTER. The F350/F330 was plotted against temperatures varying from 20°C to 95°C. Red dashed lines indicate the calculated melting temperatures (**Table 1**). **(B)** K562 cells transfected with HLA-E*0101 were loaded with the indicated peptides overnight, washed, and analyzed for their HLA-E expression. Peptide titration from three independent experiments and one representative overlay histogram and MFI are presented out of 3 independent experiments (10 µM). **(C)** NanoDSF studies to assess the thermal stability of HLA-E in complex with the following peptides: SGSDSDEEL, DSDEELVTTERKTPR, VTTERKTPR, DSDEELVTT, DMDEELVLL, and DQDEELVLL. The F350/F330 was plotted against temperature varying from 20°C to 95°C. Red dashed lines indicate the calculated melting temperatures (**Table 1**). **(D)** K562 cells transfected with HLA-E*0101 were loaded with the indicated peptides overnight, washed, and analyzed for their HLA-E expression. Peptide titration and one representative overlay histogram and MFI are presented out of 3 independent experiments (10 µM). Positive (UL40) and negative (no peptide) peptide controls are included in **(B)**. A Two-way ANOVA was used for statistical analyses of cumulative data in **(B, D)**. *Multiple comparisons were corrected by using the FDR method <0.05.

Given the rate-limiting role of HLA-E for aNK cell recognition of the pp65 peptide pool, we next sought to identify HLA-E-restricted pp65-peptides. A total of 138 15-mers from the pp65-derived peptide pool were refolded with HLA-E*0101 heavy chain and hβ_2_m, yielding a homogenous ensemble of HLA-E/peptide complexes that were isolated using size exclusion chromatography (**Supplementary Figure 2A**). All bound peptides were then eluted through mild acetic acid treatment, and the sequence identity of all bound epitopes was assessed using mass spectrometry. A total of twelve 15-mers were identified in at least two out of a total of three independent assays (**Table 2**). Later, three peptides were selected based on sequence overlap with the pp65-derived peptides identified from the HCMV strain AD169 (Inventor: Lewis L. Lanier, Patent Application Number: 16/616,435, Publication number: 20200171135). Interestingly, the sequences of pp65_85-99_ (SEVENVSVNVHNPTG), pp65_401-415_ (TSGSDSDEELVTTER), and pp65_405-419_ (DSDEELVTTERKTPR) do not comprise the classical HLA-E-restriction motifs. Instead, these three HLA-E-restricted epitopes were heavily negatively charged and contained polar residues. Molecular models of HLA-E in complex with the 15-mer peptides, pp65_85-99_, pp65_401-415_, and pp65_405-419,_ indicate that the surfaces of these complexes are significantly more electronegative compared to the surface of HLA-E in complex with classical epitopes such as UL40 or hsp60 (**Supplementary Figures 2B, C**). Molecular models of HLA-E in complex with all 9-mer peptides that we identified and designed based on the sequences and the molecular models from the three 15-mers also display the same electronegative effects on the surface of these pMHC complexes (data not shown). The capacity of each of the three identified peptides to form a complex with HLA-E and hβ_2_m was demonstrated through the successful refolding of individual HLA-E/peptide in complexes. All three obtained HLA-E/peptide complexes displayed high overall stability as measured by nano differential scanning calorimetry (nano-DSF), with melting temperature (T_m_) values stretching from 52.5 to 62°C (**Figure 3A**, **Table 1**).

**Table 2:** MS identified HLA-E-restricted pp65 peptides.

Sequence	-logP	mass	length	ppm
TPRVTGGGAMAGAST	67.41	1348.641	15	-1.4
TSGSDSDEELVTTER	65.97	1624.706	15	2.1
KAESTVAPEEDTDED	62.97	1634.679	15	-3
SEVENVSVNVHNPTG	62.95	1580.743	15	0.7
TGGGAMAGASTSAGR	56.09	1250.567	15	-0.4
TPRVTGGGAMAGAST	55.71	1332.646	15	-0.5
TVAPEEDTDEDSDNE	55.19	1664.617	15	4.6
ARNLVPMVATVQGQN	50.07	1612.836	15	2.1
EEDTDEDSDNEIHNP	48.6	1757.65	15	1.1
TLGSDVEEDLTMTRN	41.34	1695.762	15	2.6
TGGGAMAGASTSAGR	36.73	1266.562	15	0.3
DSDEELVTTERKTPR	27.67	1774.87	15	3.8

Since all three peptides induced very similar functional activities and recall responses in aNK cells compared to the HLA-E binding UL40 and Hsp60, we hypothesized that the recognition of these specific HLA-E/pp65-peptide complexes could be due to a different binding mode of NKG2C, and/or to specific properties intrinsic to the core of these particular peptides, which would promote aNK cell recall responses.

We next evaluated whether the 15-mer peptides pp65_85-99_, pp65_401-415_, and pp65_405-419_ can bind to HLA-E*0101 on target cells. The K562 cell line transfected with HLA-E*0101 was loaded overnight with the three identified peptides as well as other controls including the non-HLA-E binding H-2D^d^-restricted peptide P18-I10 (RGPGRAFVTI) (42), the HLA-E binding hsp60 peptide QMRPVSRVL, and the UL40-derived peptide VMAPRTLIL. We found that all three 15-mer peptides were able to increase the expression of HLA-E to similar levels compared to the classical UL40 and the pp65 peptide pool and at higher levels compared to all other controls (**Figure 3B**), which is well in line with the nano-DSF results.

We next addressed whether we would be able to identify 9-mer versions within the 15-mers. The three 9-mer peptides pp65_402-410_ (SGSDSDEEL), pp65_86-94_ (EVENVSVNV), and pp65_411-419_ (VTTERKTPR) were designed as potential candidates (**Table 1**), following HLA-E binding prediction by the NetMHC server. These predictions were complemented by a visual inspection of how these peptides could fit within the HLA-E binding cleft. Furthermore, as we observed a sequence overlap between pp65_402-410_ and pp65_411-419_, we also designed a fourth epitope pp65_405-413_ (DSDEELVTT). Finally, two altered peptide ligand (APL) variants, pp65_405-413_(p2Q, p9L) were designed, in which we introduced components of the HLA-E motif and therefore both predicted to bind HLA-E with a higher affinity. Refolding with pp65_402-410_, pp65_86-94_, pp65_411-419_, or with pp65_405-413_, pp65_405-413_(p2Q, p9L) and pp65_405-413_(p2M, p9L) resulted in the production of HLA-E complexes with stability that was very similar to their 15-mer counterparts as measured by nano-DSF, thus demonstrating that each nonamer could bind to HLA-E (**Figure 3C**). Furthermore, our cellular peptide binding assay revealed a similar increase in HLA-E expression levels on K562E*0101 cells loaded with pp65_402-410_, pp65_86-94_ or pp65_411-419_ as well as pp65_405-413_ and the APLs pp65_405-413_(p2Q, p9L), pp65_405-413_(p2M, p9L) (**Figure 3D**).

### 3.4 Recognition of specific HLA-E/peptide complexes by aNK cells provokes recall responses

**Figure 4:**
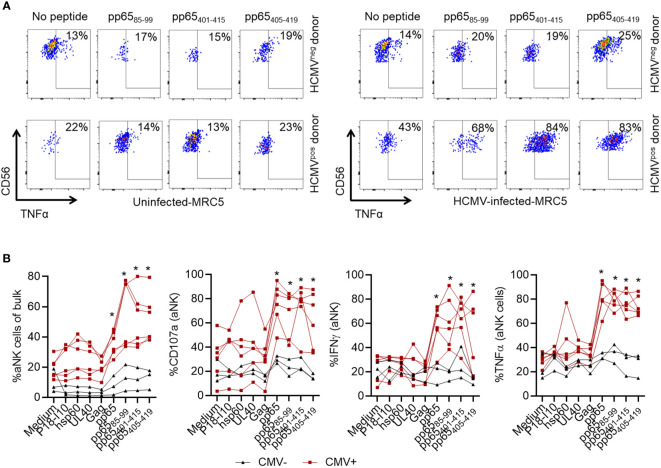
aNK cells recognize pp65-derived HLA-E-restricted 15-mer epitopes presented on mDC and perform recall responses. NK cells were cocultured with mDC in the absence of peptides or pulsed with the indicated peptides (10 µM) in the presence of 10 ng/ml IL-15 for 14 days. Later, NK cells were restimulated with HCMV-infected or uninfected MRC-5 cells before analysis of aNK cell function. Blood donors were either HCMV-seropositive (red lines) or -seronegative (black lines). Data are shown from three independent experiments and individual donors (n = 9). Representative plots **(A)** and cumulative data **(B)** are shown. A Two-way ANOVA test was used for statistical analyses and * indicating p-values ≤ 0.05.

Having demonstrated enrichment of aNK cells when in culture with mDC^+^pp65, we thereafter tested whether specific recognition of the 15-mer pp65_85-99_, pp65_401-415,_ and pp65_405-419_ epitopes could provoke recall responses by aNK cells. Therefore, NK cells were co-cultured with mDC loaded with each peptide or control peptides, including P18-I10, UL40, and Gag, or hsp60 (**Table 1**). MRC-5 cells, uninfected or infected with HCMV, were used as targets for peptide-primed aNK cells. We observed a higher TNFα production by aNK cells cultured with mDC loaded with pp65_85-99_, pp65_401-415_, or pp65_405-419_ peptides compared to unloaded mDC when restimulated with HCMV-infected MRC-5. Importantly, TNFα production was higher in aNK cells from HCMV-seropositive compared to HCMV-seronegative individuals (**Figure 4A**). Notably, following the recognition of these 15-mer peptides aNK cells displayed a marked increase in frequency, degranulation (CD107a), and cytokine production (**Figure 4B**). The observed increase in aNK cell activity was significantly higher compared to the effects of other classical HLA-E-restricted leader peptides such as UL40, and at least equal to the effects generated by the pp65-derived pool of the 15-mer peptides. Interestingly, all three pp65-derived 15-mer peptides also activated aNK cells from HCMV-seronegative individuals, yet to a much lower extent (**Figure 4B**). In contrast, the cNK cell population did not respond to the identified peptides (**Supplementary Figure 3A**). To assess the direct effect in eliciting an antigen-specific secondary response against pp65, we cultured NK cells without DC but with MRC-5 loaded with the pp65 peptide pool and assessed aNK cell responses. aNK cells displayed an increased expansion of NKG2C^+^ cells and degranulation, demonstrating a direct recognition of the pp65-derived peptides, however, to much lower levels compared to when cultured with loaded mDC (**Supplementary Figures 3B, C**). These results, suggesting that mDC are excellent presenters of these negatively charged peptides. aNK cells were subsequently cultured with mDC loaded with the nonameric peptides pp65_402-410_, pp65_86-94_, and pp65_411-419_, and our results demonstrated that at least one of these peptides elicited specific recall responses in aNK cells as measured by expansion and cytokine production (**Supplementary Figure 3D**). Hence, our results also demonstrate the equivalent capacity of 15-mer and 9-mer peptides that are heavily negatively charged to elicit such NK cell memory responses. Altogether, these results demonstrate that aNK cells can specifically recognize 15-mer HLA-E-restricted peptides, resulting in memory recall responses.

### 3.5 aNK cell antigen priming is dependent on HLA-E but not MHC class I or II

**Figure 5:**
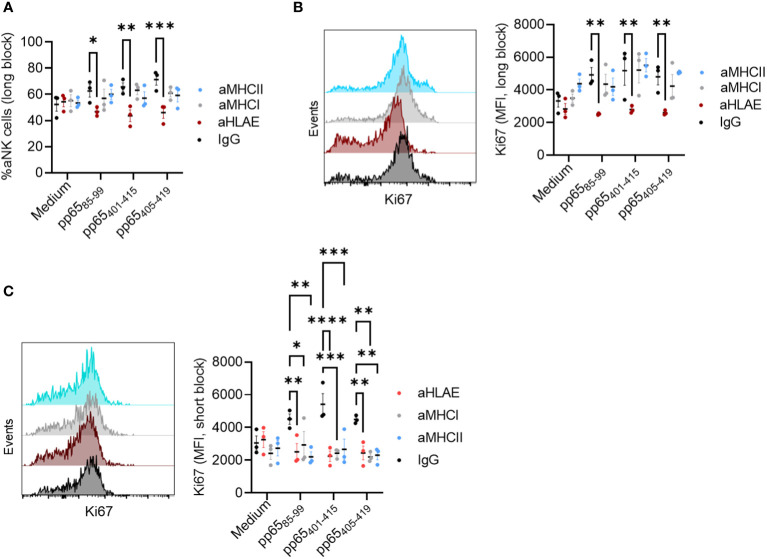
aNK cells antigen priming is dependent on HLA-E and no other MHC molecules. NK cells (n = 3) were cultured with mDC+pp65 for 14 days in the presence of either a control isotype-matched antibody IgG, or anti-HLA-E, anti-MHC I, or anti-MHC II blocking antibodies (5 μg/ml), at **(A, B)** the priming phase (day 0 and 7) or at **(C)** the restimulation phase (day 14) and thereafter assessed for their frequency and proliferation by flow cytometry. All blood donors were HCMV seropositive. The results from two independent experiments are presented, and data are shown as representative histograms for each donor. A Two-way ANOVA was used for statistical analyses. * indicating p-values ≤ 0.05, ** indicating p-values ≤ 0.001, *** indicating p-values ≤ 0.0001.

We sought to confirm that the defined 15-mer peptides are also solely presented on HLA-E and to exclude the possibility that these peptides are also presented by other MHC class I and II molecules. NK-DC was, therefore co-cultured in the presence of each of the three peptides and one of the blocking antibodies against HLA-E, MHC I, or MHC II at the priming or the restimulation phase. We found that at the priming phase, HLA-E was still the prominent antigen-presenting molecule associated with aNK cell memory (**Figures 5A, B**). On the other hand, blocking HLA-E, MHC class I, or MHC II at the secondary stimulation phase with HCMV-infected MRC-5 diminished aNK cell recall responses (**Figure 5C**). Thus, our findings suggest that aNK cell memory formation is dependent on HLA-E. However, long-term priming may result in enhanced ability of the immune cells to attack multiple epitopes on the infected cells associated with other MHC molecules than HLA-E. This phenomenon can be implied by the broad effect of MHC I, II, and HLA-E blocking at the secondary stimulation phase, which blocks the proliferation of aNK cells, giving rise to other possible mechanistic ways to target and kill the infected cells.

### 3.6 Multidimensional investigations of aNK cells confirm increased activity in response to the identified 15-mer and 9-mer peptides

**Figure 6:**
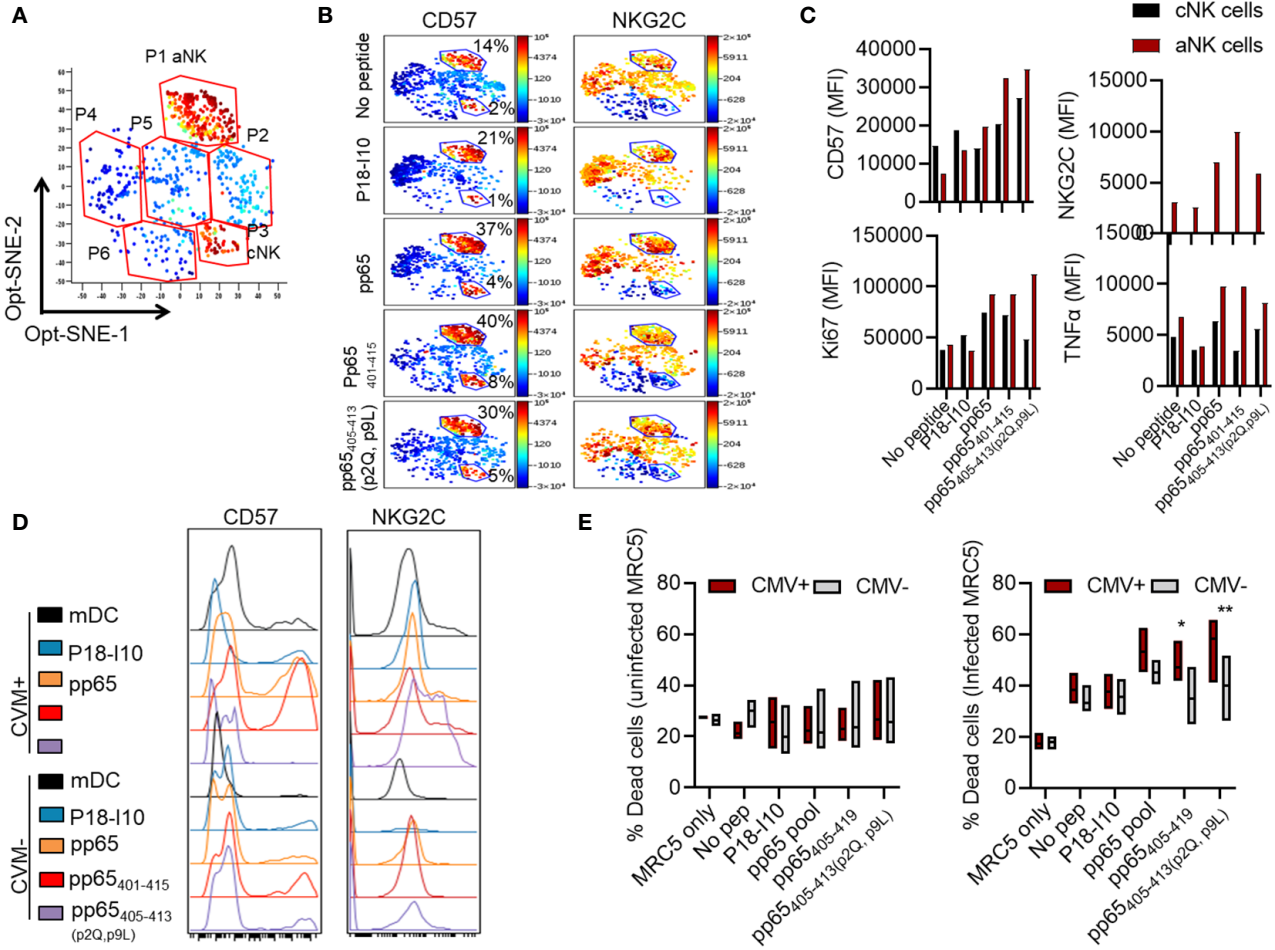
Multidimensional investigations of aNK cells confirm their specific function in response to peptides. **(A)** Dimensional reduction opt-SNE analyses of NK cells are shown following coculture with mDC loaded with pp65405-419 (DSDEELVTTERKTPR) peptide and assessed phenotypically distinct clusters. One representative opt-SNE, out of three independent experiments is shown. **(B–D)** Dimensional reduction opt-SNE analyses of NK cells are shown following coculture with mDC unloaded or loaded with different peptides and assessed for the phenotype (CD57 and NKG2C) and function (Ki67 and TNFα) of aNK and cNK cell clusters. One representative out of three independent experiments is shown. **(E)** NK cells were cultured with mDC peptide unloaded or loaded for 14 days and assessed for the killing capacity of infected or uninfected MRC-5 cells 6 hours prior to staining. Pooled data are shown from two independent experiments (n=11). Data are shown in boxplots and statistical analysis was performed using a Two-way ANOVA test * indicating p-values ≤ 0.05, ** indicating p-values ≤ 0.01.

We next sought to investigate the dynamic change of NK cell subsets following antigen priming and secondary stimulation. NK cells cultured with mDC or mDC loaded with the selected pp65 peptides were restimulated with CMV-infected MRC-5 and investigated for aNK cell identification (FcεRIγ, CD57, and NKG2C) and functional (Ki67 and TNFα) marker expression by flow cytometry. Flow cytometry data were subjected to dimensional reduction opt-SNE analysis to identify live NK cell (CD56^+^CD3^-^live/dead^-^) clusters potentially associated with specific peptide recognition. The opt-SNE analysis identified six clusters based on the markers’ expression density, including CD57, NKG2C, FcεRIγ, CD107a, Ki67, and TNFα (**Figure 6A**). These six clusters had different expression levels of the selected markers dependent on the loaded peptide (**Supplementary Figures 4**, **5A**). Among these six clusters, aNK cells and cNK cells were identified as clusters P1 and P3, respectively, based on the following characteristics: low versus high FcεRIγ, high CD57, high versus low NKG2C expression levels (**Figure 6B**, **Supplementary Figure 5A**). We found that aNK, compared to cNK cells, displayed a substantial increase in CD57 density and high expression levels of NKG2C in response to all the 15-mer and 9-mer pp65 peptides identified in this study. Functionally, these aNK cells exhibited high proliferation (presented as Ki67) and high TNFα production (**Figure 6C**), as previously shown in the 2-dimensional analysis. In addition, HLA-E-binding pp65-derived peptides, e.g., pp6_5401-415_ and pp65_405-413_(p2Q, p9L), enhanced the expression of aNK cell-associated markers CD57 and NKG2C, even in HCMV-seronegative individuals following two weeks co-culture with mDC (**Figure 6D**). Importantly, priming NK cells with pp65_405-419_ or pp65_405-413_(p2Q, p9L) resulted in specific killing of HCMV-infected compared to HCMV-uninfected MRC-5 cells (**Figure 6E**, **Supplementary Figure 5B**). In summary, our results demonstrate that aNK cells, like T cells, can form memory against HCMV-infected cells when primed with HCMV-pp65 peptide loaded DC.

## 4 Discussion

In contrast to T and B cells, NK cells have been historically regarded as phenotypically static cells with a short lifespan, unable to provide immunological MHC/peptide-specific memory. This view has been reevaluated considering more recent reports describing the phenotypic, epigenetic, and functional heterogeneity that exists among populations of NK cells, particularly in response to viral infections (10, 43–45). In the present study, we demonstrated that aNK cells can recognize 15-mer HLA-E-restricted HCMV peptides with unconventional sequences, and establish a memory response resulting in a clonal-like expansion following a secondary stimulation. This peptide-specific recognition elicited significant recall responses that led to the killing of HCMV-infected targets, thus confirming their unambiguous immunological memory of specific epitopes.

Recent discoveries established that aNK cells are able to respond to specific viral antigens through interaction with antigen-loaded non-polymorphic HLA-E (3). Cell-surface stabilization of HLA-E requires loading with peptides, which can be derived from MHC class I leader peptides or other proteins at steady state (46–48). In addition to host peptides, the UL40 motif in HCMV has been found to encode HLA-E-stabilizing peptides that are partially similar to MHC class I leader sequences (49, 50). Here we show that none of the three peptides comprised the classical HLA-E-restriction motifs, which include a methionine residue that binds to the HLA-E B-pocket or a leucine/valine residue at p9 that would fit in the hydrophobic F-pocket of HLA-E (51, 52). Furthermore, none of these three peptides contain glutamine or a lysine residue that could be used as anchor positions for binding to the B-pocket, as described in HLA-E epitope mapping studies (52, 53), or proline residues at p3, p4, p6 or p7, all shown to enhance the binding capacity of peptides to HLA-E (54). Instead, these three HLA-E-restricted epitopes were all heavily negatively charged and comprised polar residues. None of these three epitopes was predicted to bind to HLA-E by the MHC peptide prediction server NetMHC (55). However, it should be noted that parts of these three peptide sequences have been previously described/predicted in the literature mainly as targets for B cell recognition (56, 57) or as epitopes restricted to classical MHC class I and class II molecules (58–60). To our knowledge, all the previously determined crystal structures of HLA-E present nonameric peptides, which bind stably to HLA-E. However, recent MS analyses have demonstrated that nonameric peptides constitute only 18% of the whole HLA-E immunopeptidome and demonstrated the presence of longer peptides with lengths ranging from 10 to 21 amino acids (53, 61). Interestingly, although the identified peptide sequences do not have homology with the traditional motif, they bind to HLA-E*0101, as demonstrated by both molecular and cellular binding assays. In addition, all three 15-metric peptides identified within the present study induced recall responses in aNK cells that could be due to specific molecular features, including their significant electronegativity. Indeed, similar to our negatively charged 15-mer HCMV peptides, some of the peptides derived from *M. tuberculosis* proteins were acidic and were recognized by CD8 T cells (61).

HCMV-infected cells and several solid tumors overexpress HLA-E as an escape mechanism of cNK and T cell killing through ligation of the inhibitory receptor NKG2A (38, 50, 62). Theoretically, inhibition of NKG2A will allow for the interaction of the NKG2A counterpart, the activating receptor NKG2C with HLA-E and may allow for specific targeting. A phase II clinical trial in which anti-NKG2A was combined with an epidermal growth factor receptor inhibitor in previously treated head and neck carcinomas showed a 31% objective response rate (63). Hypothetically, these clinical responses might be due to the NK cell activation status or tumor-antigen recognition by aNK cells, and whether the patient is an HCMV carrier. Here, we found that inhibition of NKG2A in aNK cells interacting with mDC loaded with HCMV-peptides did not change their activation state, excluding the possibility that the identified peptides bind to NKG2A. In contrast, antibody blocking of HLA-E and NKG2C interactions significantly altered the activation state of aNK cells in coculture with peptide-loaded mDC, indicating an antigen recognition state through HLA-E/peptide/NKG2C complexes rather than a co-stimulation boost.

Here, we hypothesized that similar to T cells, the activation of aNK cells may involve both antigen recognition (signal 1) and co-stimulatory signals combined with signaling from cytokines provided by professional antigen-presenting cells (APC) (signal 2 and 3). We and others have previously demonstrated that both DC and B cells can activate NK cells (64–67). However, whether APC are also essential for aNK cells to display a secondary immune response was unknown. Our results reveal that aNK cells depend on at least signal 1. Further studies are needed to determine the specific DC co-stimulatory receptors (signal 2) and cytokine stimulation for aNK cell recall responses and memory persistence.

Our results have implications for strategies to expand aNK cells with immunological memory *ex vivo* for immunotherapy. Several transplantation studies have shown that NK cells are involved in tumor rejection and protection from relapse, supporting the therapeutic potential of NK cells in tumor eradication (68, 69). Recently, it has been shown that aNK cells with single-KIR+NKG2C+ expanded from selected HCMV infected donors with feeder cells loaded with HLA-G leader-derived peptides have potent reactivity towards HLA-mismatched acute myeloid leukemia cells (4). Despite these encouraging findings, NK cell therapies are limited by the lack of antigen specificity. Also, similar to T cells, resistance to NK cell-mediated killing may also develop due to the recruitment and differentiation of immune suppressive cells, including regulatory T cells (Treg) and myeloid-derived suppressor cells (MDSC), as well as overexpression of immune inhibitory checkpoint proteins in the tumor microenvironment (TME). We discovered earlier that aNK cells found in HCMV-seropositive individuals can resist TME-induced suppression. We showed that the mechanisms sparing aNK cells from immune suppression by MDSC and Treg involved the downregulation of the checkpoint molecules T cell immunoglobulin and ITIM domain (TIGIT), programmed death receptor (PD-1) and IL-1R8 (22, 23). In agreement with these findings, we found that reconstitution and expansion of aNK cells in individuals with HCMV reactivation was associated with reduced leukemia relapse and better clinical outcomes following hematopoietic stem cell transplantation (22, 24). Thus, our 15-mer identified peptides could potentially be used as a therapeutic vaccine strategy to provoke antigen-specific aNK cell responses with persistent memory, combined with the ability to resist immunosuppression. Ongoing studies in our lab evaluate the potential of this strategy in solid tumors.

## Data availability statement

The raw data supporting the conclusions of this article will be made available by the authors, without undue reservation.

## Ethics statement

The studies involving humans were approved by Karolinska Institutet institutional review board. The studies were conducted in accordance with the local legislation and institutional requirements. The participants provided their written informed consent to participate in this study.

## Author contributions

NA and BS performed experiments analyzed data, visualization, methodology, writing and editing manuscript. TS, YS, TR, FC performed experiments, collected data, formal analysis, reviewing manuscript. CS-N, JM, AA. Provided study materials, reagents, instrumentation, formal analysis, or other analysis tools, validation, supervision, editing, and revising manuscript. DS Conceptualization, formal analysis, supervision, funding acquisition, validation, investigation, visualization, methodology, writing and editing manuscript, project administration. All authors contributed to the article and approved the submitted version.
