# Supplementary material for: Non-classical HLA-E restricted CMV 15-mer peptides are recognized by adaptive NK cells and induce memory responses
Source: Front Immunol. 2023 Sep 21;14:1230718. doi: 10.3389/fimmu.2023.1230718 (PMC10552778; doi:10.3389/fimmu.2023.1230718)
Supplement: Supplementary file 1 [file DataSheet_1.pdf]

frontiers

Supplementary Material

Non-classical HLA-E restricted CMV 15-mer peptides are recognized by adaptive NK cells and induce memory responses

Nerea Martín Almazán[1]†, Benedetta Maria Sala[2]†, Tatyana Sandalova[2], Yizhe Sun[1], Tom Resink[2], Frank Cichocki[3], Cecilia Söderberg-Nauclér[4,5], Jeffrey S. Miller[3], Adnane Achour[2], Dhifaf Sarhan[1*]

[1]Karolinska Institute, Department of Laboratory Medicine, Division of Pathology, Stockholm, Sweden

[2]Karolinska Institute, Department of Medicine Solna, Division of Infectious Diseases, Karolinska University Hospital, Stockholm, Sweden

[3]University of Minnesota Masonic Cancer Center, Division of Hematology, Oncology and Transplantation, Minneapolis, Minnesota, USA

[4]Karolinska Institute, Department of Medicine, Microbial Pathogenesis Unit, Stockholm, Sweden

[5]Karolinska University Hospital, Division of Neurology, Stockholm, Sweden

*** Correspondence:**
Corresponding Author: Dhifaf Sarhan, Karolinska Institute, Department of Laboratory Medicine, Division of Pathology, Stockholm, Sweden, Phone: +46704487830; Email: dhifaf.sarhan@ki.se

† These authors contributed equally to this work and share first authorship.

1 Supplementary Figures

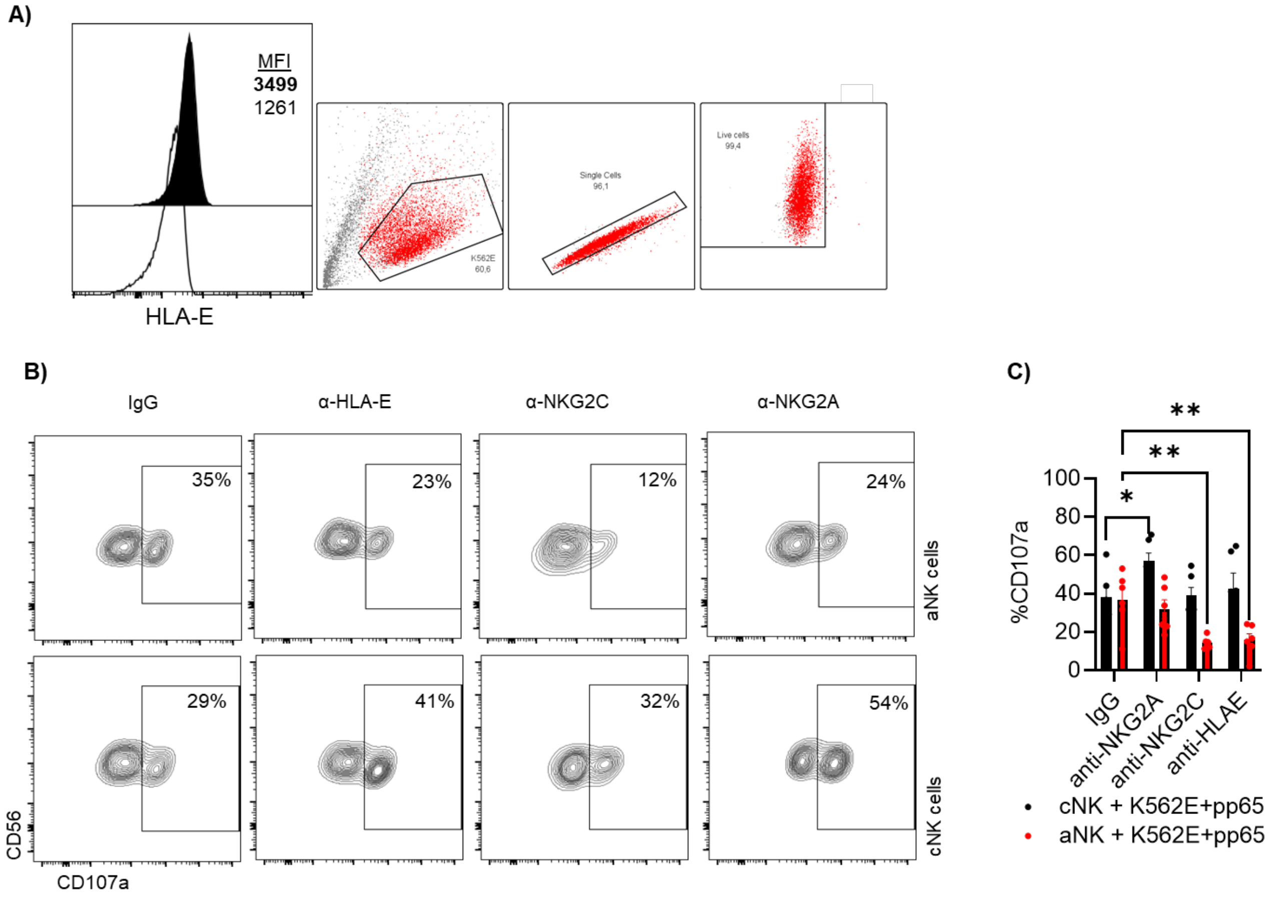

Supplementary Figure 1. A) Original K562 and K562 cells transfected with HLA-E*0101 were analyzed for their HLA-E expression. One representative histogram of K562 (empty) K562*0101 (filled) with no peptide and gating strategy is shown. **B)** Representative or **C)** accumulative data are shown of NK cells (n = 6) cultured with K562 cells transfected with HLA-E, loaded with pp65, in the presence of either a control isotype-matched antibody IgG, anti-HLA-E, anti-NKG2C, or anti-NKG2A blocking antibodies (5 µg/ml), and thereafter assessed for cNK and aNK cell degranulation by flow cytometry. The data are shown as representative plots. Multiple comparison test was used for statistical analyses, and * indicating p-values ≤ 0.05 and ** ≤ 0.005.

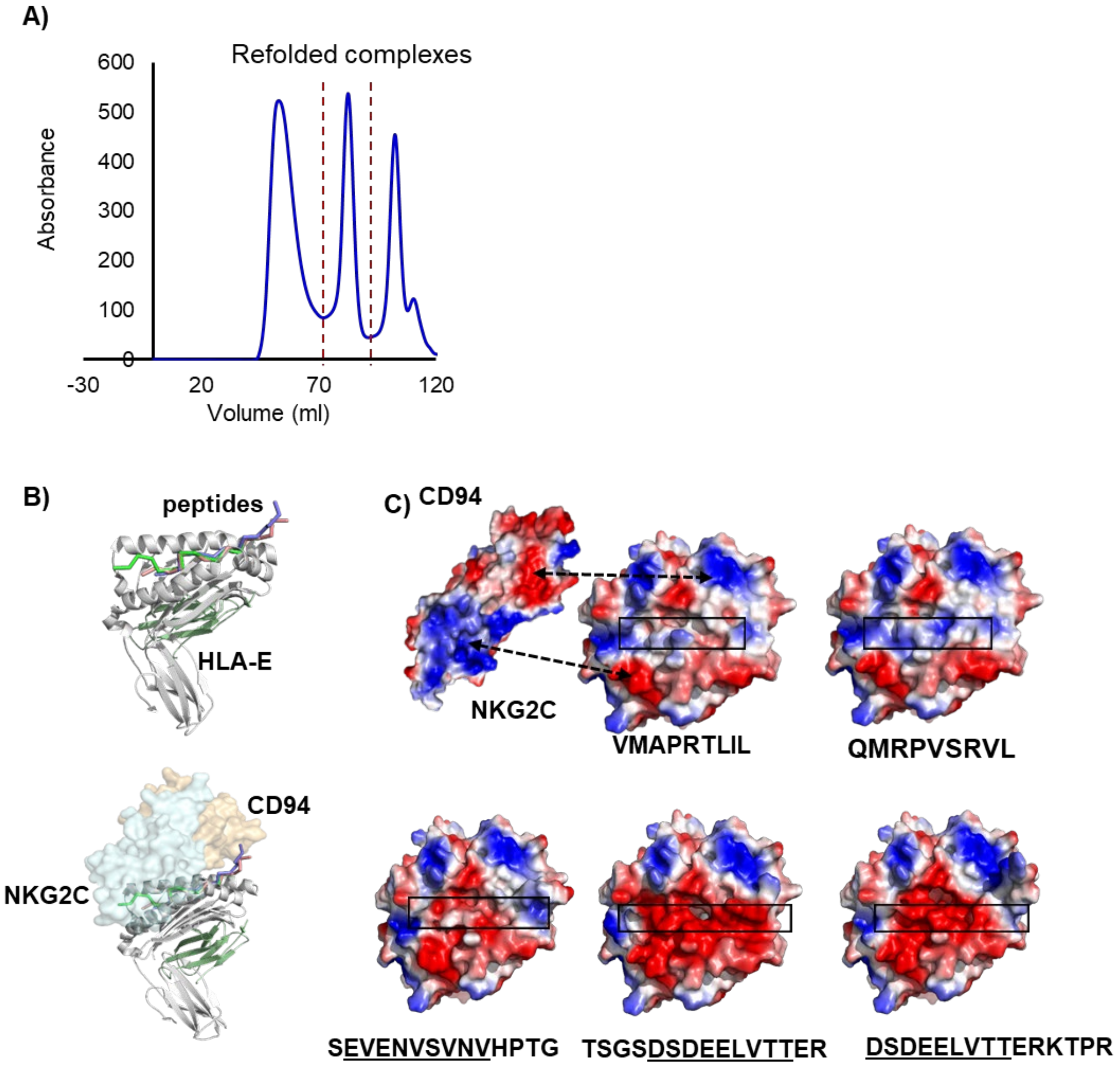

Supplementary Figure 2. A) Size exclusion chromatogram of HLA-E refolded in the presence of the pp65 peptide pool. Red-dashed lines indicate the fraction that was pooled to obtain correctly refolded complexes. **B)** Hypothetical molecular models of HLA-E in complex with pp65$_{85-99}$ (SEVENVSVNVHNPTG, in pink), pp65$_{401-415}$ (TSGSDSDEELVTTER, in green) or pp65$_{405-419}$ (DSDEELVTTERKTPR, in blue). **C)** A molecular model of the NKG2C/CD49 heterodimer is presented with arrows that indicate the positively (in blue) and negatively (in red) charged sections that may interact with specific regions on the previously determined crystal structures of HLA-E in complex with classical epitopes such as UL40 (VMAPRTLIL) or hsp60 (QMRPVSRVL). A rectangular frame indicates the position of each bound peptide. The lower panel displays the hypothetical molecular models of HLA-E in complex with the three 15-mer peptides identified within this study pp65$_{85-99}$ (SEVENVSVNVHNPTG), pp65$_{401-415}$ (TSGSDSDEELVTTER) and pp65$_{405-419}$ (DSDEELVTTERKTPR). It should be noted that only the assumed nonameric cores of each 15-mer peptide (underlined in the figure) were modeled.

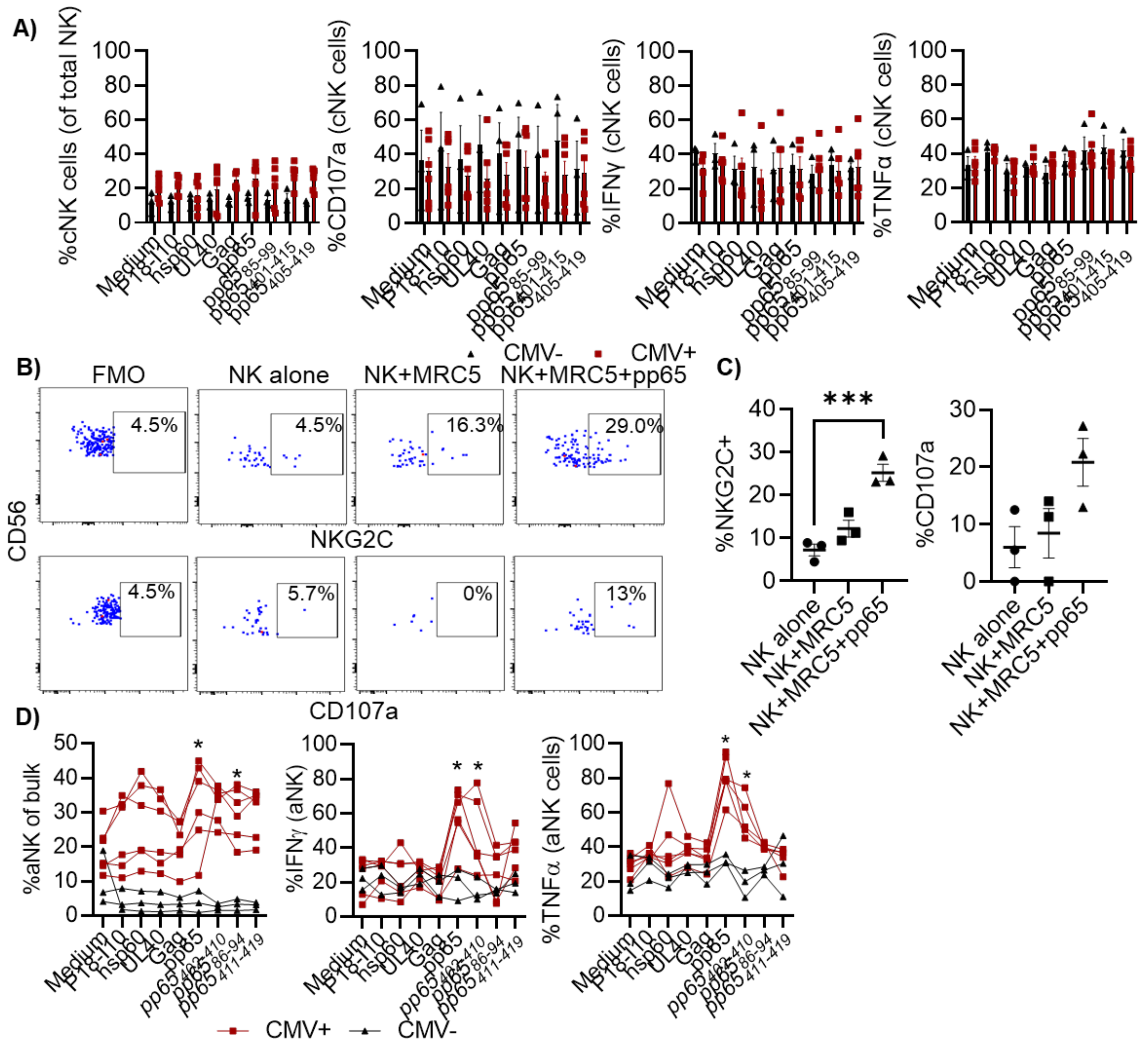

Supplementary Figure 3. A) NK cells were cocultured with mDC without peptides or loaded with the indicated peptide (10 µM) in the presence of 10 ng/ml IL-15 for 14 days. Later, NK cells were restimulated with HCMV-infected MRC-5 cells before analysis of cNK cell function. **B)** After 10 days of culture in IL-15, NK cells were cultured with MRC-5 in the presence or absence of pp65 and assessed for NKG2C frequency and CD107a following 6 hours of stimulation prior to staining. One representative experiment and **C)** accumulative data are shown of a total of 3 independent, and One-way ANOVA test was used for statistical analyses and *** indicating p-values ≤ 0.001. **D)** NK cells were cocultured with mDC without peptides or pulsed with the indicated peptides (10 µM) in the presence of 10 ng/ml IL-15 for 14 days. Later, NK cells were restimulated with HCMV-infected prior to analysis of aNK cell function. Blood donors were either HCMV-seropositive or -seronegative. Data are shown from three independent experiments and individual donors (n = 9). Individual donor's data are displayed. A two-way ANOVA test was used for statistical analyses and * indicating p-values ≤ 0.05.

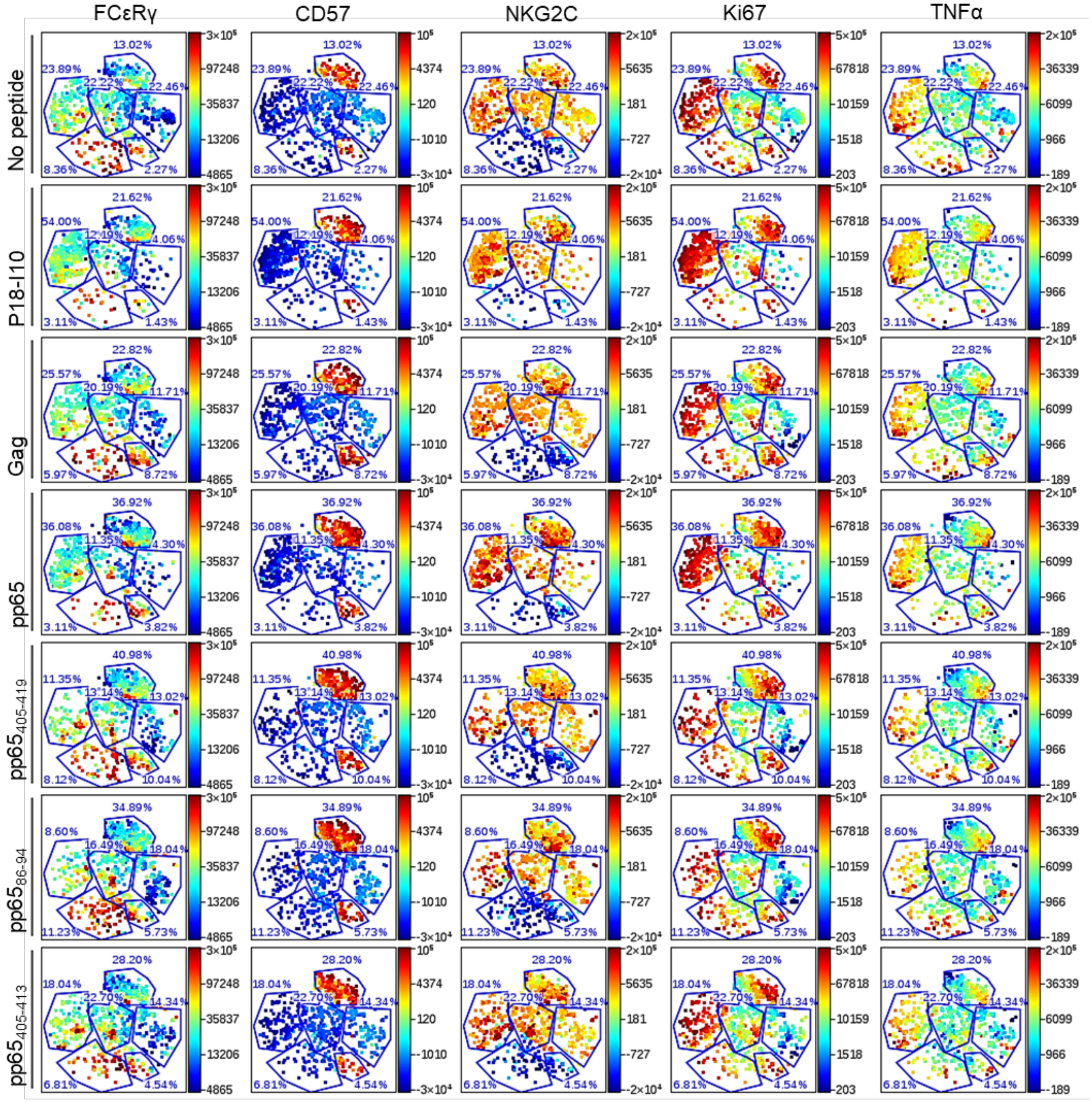

Supplementary Figure 4. Multidimensional investigations reveal six different NK cell populations in response to the discovered peptides. Dimensional reduction analysis of NK cells following coculture with mDC unloaded or loaded with other peptides. One representative experiment is shown of 3 independent experiments.

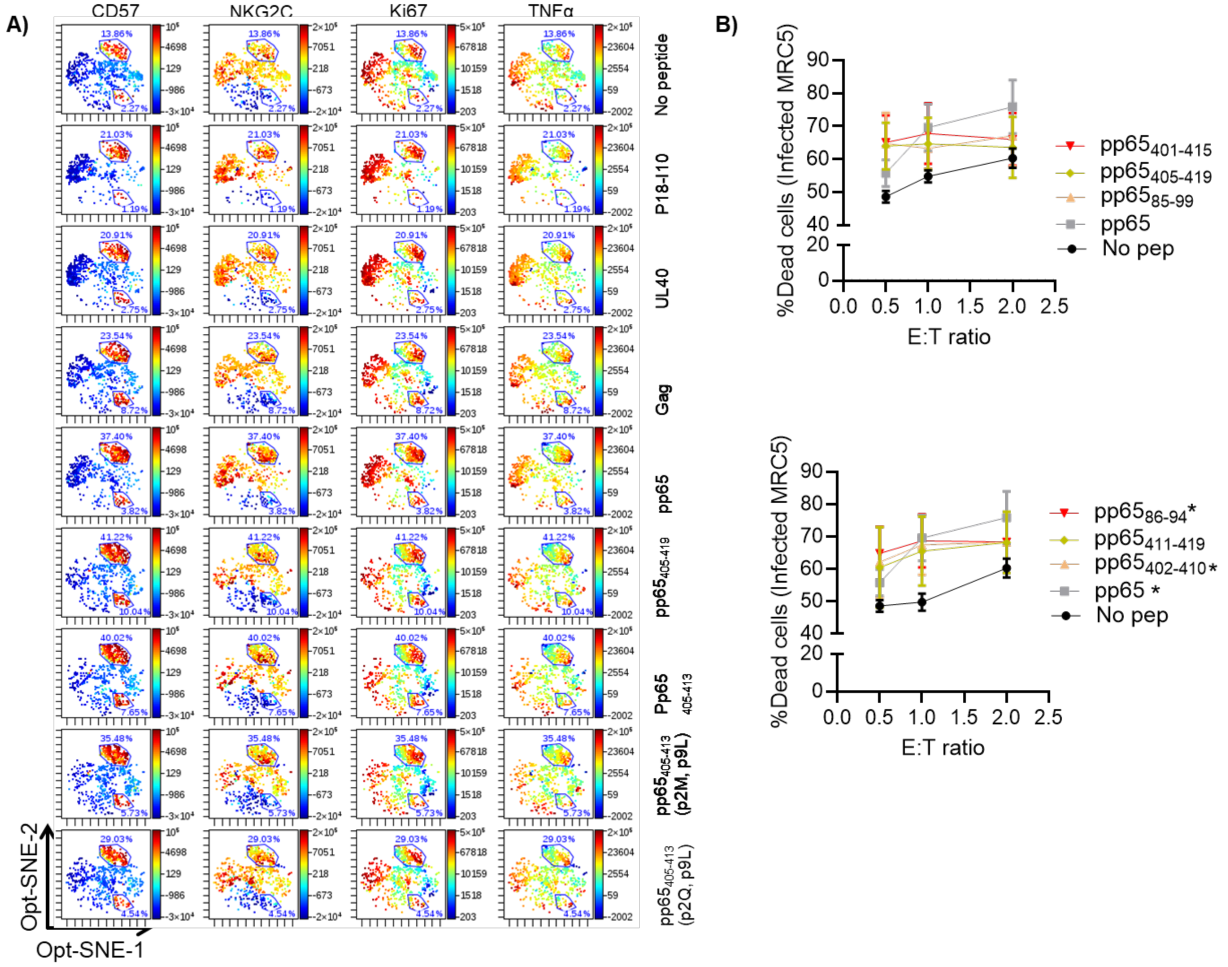

Supplementary Figure 5. Multidimensional investigations of aNK cells confirm their specific function in response to the discovered peptides. A) Dimensional reduction analysis of NK cells following coculture with mDC unloaded or loaded with different peptides, including modified peptides. One representative experiment is shown of 3 independent experiments. **B)** NK cells were cocultured with mDC without peptides or pulsed with the indicated peptides (10 µM) for 14 days. Then, NK cells were cocultured with HCMV-infected MRC-5 cells at the indicated E: T ratios. A two-way ANOVA test was used for statistical analyses, and * indicating p-values ≤ 0.05 calculated on an E:T ratio of 1:1.
